# Genetic screening and functional characterization of *DUOX2* mutations in patients with congenital hypothyroidism

**DOI:** 10.1210/jendso/bvag188

**Published:** 2026-08-27

**Authors:** Su-Hong Huang, Feng Sun, Fei Luo, Hui-Lan Li, Hai-Lin Huang, Jing-Min Guo, Chong Guo, Xue-Hua Zhang, Shuang-Xia Zhao, Bo-Kun Wei, Huai-Dong Song, Rui Li, Feng Cheng

**Affiliations:** Department of Clinical Laboratory, Fujian Children's Hospital (Fujian Branch of Shanghai Children's Medical Center), College of Clinical Medicine for Obstetrics & Gynecology and Pediatrics, Fujian Medical University, Fuzhou, Fujian Province 350014, China; Department of Endocrinology, The Core Laboratory in Medical Center of Clinical Research, Shanghai Ninth People's Hospital, Shanghai Jiao Tong University School of Medicine, Shanghai 200011, China; Department of Clinical Laboratory, Fujian Children's Hospital (Fujian Branch of Shanghai Children's Medical Center), College of Clinical Medicine for Obstetrics & Gynecology and Pediatrics, Fujian Medical University, Fuzhou, Fujian Province 350014, China; The School of Medical Technology and Engineering, Fujian Medical University, Fuzhou, Fujian Province 350004, China; Department of Clinical Pathology, Shaowu Municipal Hospital, Shaowu, Fujian Province 354000, China; Department of Child Healthcare, Fujian Maternity and Child Health Hospital, College of Clinical Medicine for Obstetrics & Gynecology and Pediatrics, Fujian Medical University, Fuzhou, Fujian Province 350001, China; Department of Child Healthcare, Fujian Maternity and Child Health Hospital, College of Clinical Medicine for Obstetrics & Gynecology and Pediatrics, Fujian Medical University, Fuzhou, Fujian Province 350001, China; Department of Ultrasound, Fujian Children's Hospital (Fujian Branch of Shanghai Children's Medical Center), College of Clinical Medicine for Obstetrics & Gynecology and Pediatrics, Fujian Medical University, Fuzhou, Fujian Province 350014, China; Department of Endocrinology, The Core Laboratory in Medical Center of Clinical Research, Shanghai Ninth People's Hospital, Shanghai Jiao Tong University School of Medicine, Shanghai 200011, China; The School of Medical Technology and Engineering, Fujian Medical University, Fuzhou, Fujian Province 350004, China; Department of Endocrinology, The Core Laboratory in Medical Center of Clinical Research, Shanghai Ninth People's Hospital, Shanghai Jiao Tong University School of Medicine, Shanghai 200011, China; Department of Endocrinology, The Core Laboratory in Medical Center of Clinical Research, Shanghai Ninth People's Hospital, Shanghai Jiao Tong University School of Medicine, Shanghai 200011, China; Department of Clinical Laboratory, Fujian Children's Hospital (Fujian Branch of Shanghai Children's Medical Center), College of Clinical Medicine for Obstetrics & Gynecology and Pediatrics, Fujian Medical University, Fuzhou, Fujian Province 350014, China

**Keywords:** congenital hypothyroidism, *DUOX2*, enzymatic activity, genotype–phenotype analysis

## Abstract

**Context:**

Congenital hypothyroidism (CH) is the most prevalent endocrine disorders detected via newborn screening. Variants in dual oxidase 2 (*DUOX2*) have been established as a major genetic contributor to CH in Chinese populations.

**Objective:**

To characterize the genetic spectrum of *DUOX2* variants and explore their clinical correlations in a cohort of CH patients from Fujian Province, China, and to assess variant impact on enzymatic activity.

**Methods:**

We enrolled 184 Han Chinese CH patients. *DUOX2* variants were screened via exome sequencing. Clinical data (thyroid function, ultrasonography) were integrated with in vitro measurement of H_2_O_2_ generation for selected variants.

**Results:**

Of the 184 patients, 114 (61.96%) carried genetic variants. *DUOX2* was the most prevalent, accounting for 43.48% (80/184) of cases. Notably, statistically significant differences in TSH, FT4, and FT3 levels were observed between *DUOX2* variant carriers and noncarriers. Ultrasonography revealed that normal thyroid morphology was the predominant finding in children with *DUOX2* variants. In vitro functional assays revealed that 5 out of 11 *DUOX2* variants exhibited significantly impaired H_2_O_2_ production capacity. Notably, variants in both functional and unstructured domains impaired residual enzymatic activity.

**Conclusion:**

*DUOX2* is the most common causative gene for CH in the Fujian cohort, and its variants are associated with a more severe biochemical phenotype at onset diagnosis. Functional heterogeneity suggests that impaired enzymatic activity is a key, but not exclusive, pathogenetic mechanism. Ultimately our findings highlight the critical need for integrating genetic and functional analyses into the clinical management and genetic counseling of CH.

Congenital hypothyroidism (CH) is a common pediatric endocrine and metabolic disorder characterized by defects in thyroid hormone synthesis, secretion, or receptor function, affecting approximately 1 in 2000 to 4000 live births worldwide (1, 2). Congenital hypothyroidism can lead to irreversible intellectual disability and growth retardation; however, timely neonatal screening and standardized levothyroxine replacement therapy can significantly mitigate these adverse outcomes (3).

Genetic sequencing studies have identified substantial ethnic and geographic variation in the prevalence of genes associated with CH. In particular, mutations in the thyroglobulin (*TG*) and thyroid peroxidase (*TPO*) genes are more commonly detected in Western populations (4-6). In contrast, mutations in the dual oxidase 2 (*DUOX2*) represent the most common genetic cause of CH in Asian countries, including China (7-10). The *DUOX2*, located on chromosome 15q21.1, encodes a nicotinamide adenine dinucleotide phosphate (NADPH)-dependent oxidase. This enzyme is primarily expressed in the apical membrane of thyroid follicular cells, where it plays a critical role in thyroid hormone synthesis by generating hydrogen peroxide (H_2_O_2_)—an essential substrate for the TPO-mediated iodination of tyrosine residues in TG (11, 12). *DUOX2* variants have been associated with both transient and permanent CH, with striking phenotypic heterogeneity observed even among patients carrying the same variant (13-15). Previous studies have reported that *DUOX2* variants can cause not only impaired thyroid hormone synthesis but also structural thyroid abnormalities such as goiter or ectopic thyroid (11, 12). Furthermore, functional studies have demonstrated that pathogenic *DUOX2* variants reduce H_2_O_2_ generation capacity, thereby disrupting thyroid hormone biosynthesis (16, 17). However, due to population genetics, the mutation spectrum of *DUOX2*, its correlation with clinical phenotypes, and the resulting functional impacts may significantly across different regions of China.

Although genetic sequencing data for CH patients have been widely reported across different regions of China, few studies have focused specifically on how gene mutations correlate with clinical phenotypes (18-20). In our research, we aimed to systematically analyze the genetic mutation spectrum of CH patients from Fujian, and to explore the links between mutation types and clinical features—including thyroid function tests, thyroid ultrasound findings, physical growth, and neurodevelopmental outcomes. Additionally, we performed in vitro functional analyses on selected *DUOX2* variants to further assess their pathogenic potential.

## Materials and methods

### Patients

A total of 184 blood samples were collected from children with complete clinical data between 2020 and 2024. The screening, diagnosis, and treatment for CH were based on the Congenital Hypothyroidism Diagnosis and Treatment Consensus established by the Chinese Medical Association. Patients exhibiting TSH levels of ≥10 mU/L during the initial screening were classified as positive cases which were recalled for re-examination using either an immunochemiluminescence assay (RRID:AB_3675942 ADVIA Centaur® FT3 assay; RRID:AB_2783805 ADVIA Centaur® CP FT4; RRID:AB_2923011 TSH3-Ultra (TSH3-UL)) to assess serum TSH, free triiodothyronine (FT3), and free thyroxine (FT4) levels. Exclusion of acquired thyroid disorders, maternal thyroid disease, and other congenital syndromes associated with CH. The median age at diagnosis was 20 days (interquartile range: 10-30 days). We systematically collected clinical data, including thyroid function tests and thyroid ultrasonography at disease onset, along with assessments of physical growth and neurodevelopment during follow-up. Growth and neurodevelopmental assessment data were derived from the Wechsler Intelligence Scale, the Gesell Developmental Schedules, and the Child Neuropsychological Development Checklist II. This study was approved by the Ethics Committee of a tertiary children's hospital (approval no. 2022ETKLR0113 and 2024ETKLRK09016). Written informed consent was obtained from the parents or legal guardians of all participants.

### Exome sequencing

Exome sequencing was performed as previously described (21), with minor modifications. Genomic DNA was extracted from peripheral blood samples of the probands using the Quick Gene DNA Whole Blood Kit L (Kurabo, Japan) in accordance with the manufacturer's instructions. The extracted DNA was fragmented to a size range of 200 to 300 bp via sonication. Subsequently, barcoded sequencing adapters were ligated to the fragmented DNA using the KAPA HyperPrep Kit (Roche, Switzerland). Target genomic regions were enriched via hybridization capture using the Roche SeqCap EZ Library, according to the manufacturer's protocol (Roche SeqCap EZ Library SR User's Guide). Paired-end sequencing was conducted on an Illumina HiSeq 3000 platform with a read length of 150 bp, achieving an average sequencing depth of approximately 100×. We applied the classification criteria established by the American College of Medical Genetics and Genomics to categorize the identified genetic variants into 5 classes: pathogenic, likely pathogenic, variants of uncertain significance (VUS), likely benign, and benign (22). Only pathogenic, likely pathogenic, and VUS variants in genes previously associated with CH were included in subsequent analyses.

### Cell transfection and in vitro functional analysis of DUOX2 variants

Human wild-type (WT) DUOXA2-myc/His (with C-terminal myc/His tags) was cloned into the pcDNA3.1 expression vector (DUOXA2-myc/His-pcDNA3.1). All constructs were verified by Sanger sequencing. 293-T cells were seeded in 24-well plates. After cells reached 60% to 70% confluence, either WT or mutant HA-DUOX2-pEGFP-N2 expression plasmids were co-transfected with WT DUOXA2-myc/His-pcDNA3.1 vector into 293T cells using X-tremeGENE HP DNA Transfection Reagent (Roche Applied Science, Indianapolis, IN, USA) following the manufacturer's protocol. 48 hours post-transfection, the cell monolayers were incubated with 1 µM ionomycin (Sigma, St. Louis, MO, USA) in Dulbecco's PBS supplemented with 50 mM Amplex Red reagent and 0.1 U/mL horseradish peroxidase (Sigma, St. Louis, MO, USA) for 1 hour at 37 °C. Relative fluorescence units (excitation/emission 530/590 nm) of the medium were measured within the linear range of the H_2_O_2_ concentration response curve. Absolute H_2_O_2_ concentrations (nanomoles) were calculated using a calibration curve. The enzymatic activity of each mutant was expressed as a percentage of the WT activity. All experiments were performed in triplicate.

### Statistical analyses

Statistical analyses were conducted using SPSS version 25.0 (IBM Corp, Armonk, NY, USA) and GraphPad Prism 9 (GraphPad Software, San Diego, CA, USA). The Shapiro–Wilk test was used to assess the normality of continuous variables. Non-normally distributed data are presented as the median (interquartile range, IQR), and intergroup comparisons were performed utilizing the Kruskal–Wallis H test, with post hoc pairwise comparisons with Bonferroni correction. Categorical variables were compared using the χ^2^ test or Fisher exact test. A 2-sided *P*-value of ≤0.05 was considered statistically significant.

## Results

### Variant patterns in 184 patients with CH

Exome sequencing identified 114 of 184 CH patients (61.96%) carrying at least 1 variant among 21 CH-associated genes. These genes included *DUOX2*, *TG*, *DUOXA1*, *TPO*, *DUOXA2*, *DUOX1*, *DIO2*, *DIO3*, *GNAS*, *SLC26A4*, *GLIS3*, *TSHR*, *SLC26A7*, *IYD*, *SLC16A2*, *FOXE1*, *GBP1*, *HHEX*, *ISL1*, *JAG1*, and *SHH* (21, 23-25). *DUOX2* variants were the most frequent, detected in 80 patients (43.48%), while 34 patients cases had variants in other genes and did not carry any *DUOX2* variants. Variants in rarely reported genes (eg, *DIO2*, *DIO3*, *GNAS*) were found as isolated alterations in 8 patients (4.35%), and co-occurred with *DUOX2* variants in 23 patients (12.5%). In line with recent large-scale Chinese CH screening studies, *DUOXA2* variants often coexisted with *DUOX2* variants, supporting an oligogenic pattern of the DUOX system in CH pathogenesis (26).

**Figure 1:**
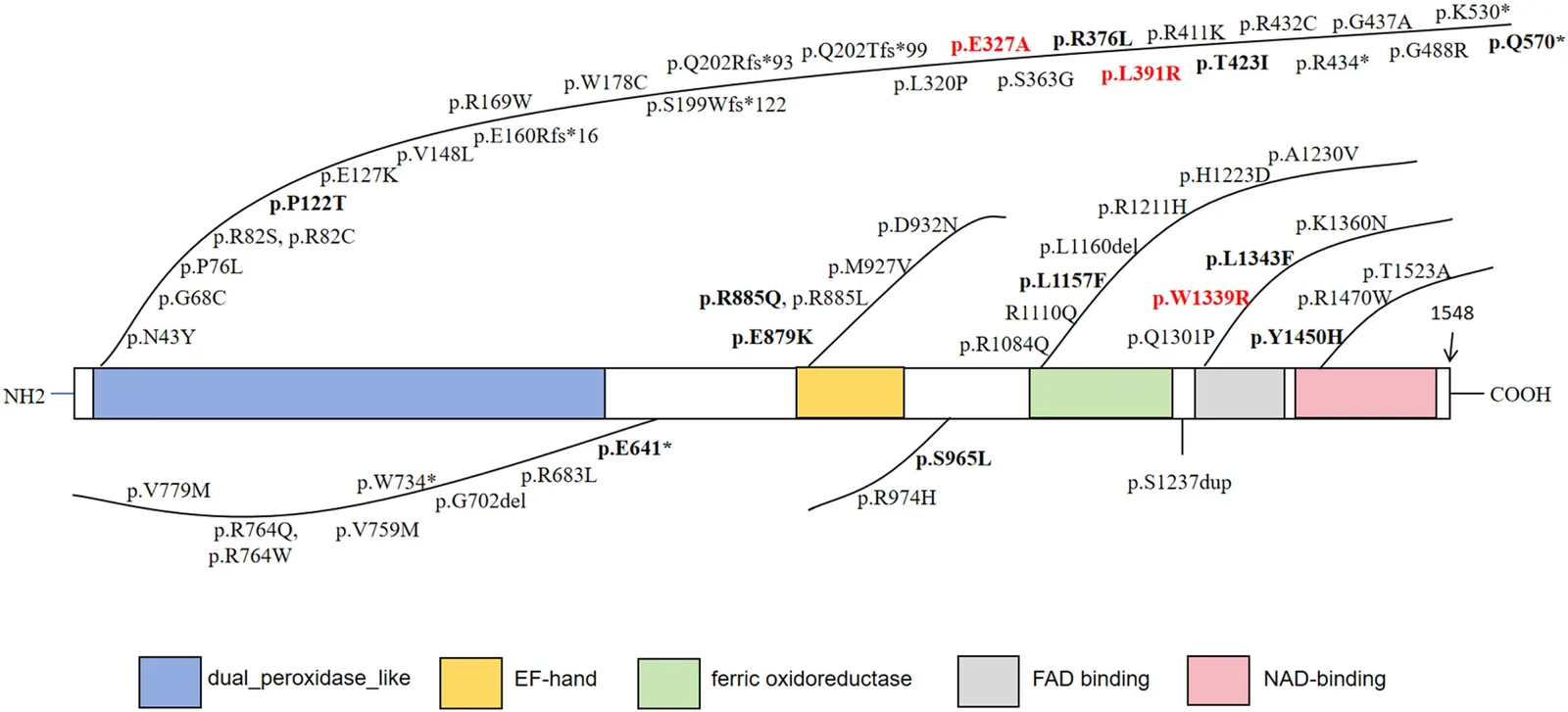
Distribution of *DUOX2* variants in patients with congenital hypothyroidism (CH). The linear map of DUOX2 protein shows 5 functional domains with their corresponding amino acid (aa) positions: dual peroxidase-like domain (blue, 38-594 aa), EF-hand domain (yellow, 819-934 aa), ferric oxidoreductase domain (green, 1084-1233 aa), FAD-binding domain (gray, 1271-1369 aa), and NAD-binding domain (pink, 1377-1531 aa). The horizontal scale bar represents the full-length of DUOX2 protein (1-1548 aa). All variants are labeled following HGVS nomenclature. Red bold: novel variants identified in this study; black bold: mutation types in functional assays.

Among the identified *DUOX2* variants, the most frequent variant was c.1588A > T (p.K530*) (*n* = 35), followed by c.3329G > A (p.R1110Q) (*n* = 22), c.4027C > T (p.L1343F) (*n* = 15), c.2048G > T (p.R683L) (*n* = 14), c.3693 + 1G > T (*n* = 13), c.2654G > T (p.R885L) (*n* = 9) (Table 1). A total of 63 distinct variant sites were identified within the *DUOX2*. Sixty have been previously documented, while the remaining 3 are novel: c.980A > C (p.E327A), c.4015T > C (p.W1339R), and c.1172T > G (p.L391R). These 63 variants were distributed across different domains of DUOX2: 27 variants were located in the dual peroxidase-like domain, 5 in the EF-hand domain, 7 in the ferric oxidoreductase domain, 4 in the FAD-binding domain, 3 in the NAD-binding domain, and 11 in the unstructured domain, additionally 6 variants were found in the noncoding regions (Fig. 1).

**Table 1:** The 63 *DUOX2* variants identified in our study

No.	Variant sites	Variant types	cDNA change	Amino acid change	Classification	Number of patients	SNP ID
1	Exon14	Nonsense	c.1588A > T	p.K530*	Pathogenic	35	rs180671269
2	Exon25	MISSENSE	c.3329G > A	p.R1110Q	Pathogenic	22	rs368488511
3	Exon30	MISSENSE	c.4027C > T	p.L1343F	Pathogenic	15	rs147945181
4	Exon17	MISSENSE	c.2048G > T	p.R683L	Pathogenic	14	rs8028305
5	Exon28	Intronic splicing	c.3693 + 1G > T	—	LP	13	rs200717240
6	Exon20	Missense	c.2654G > T	p.R885L	Pathogenic	9	rs181461079
7	Exon20	Missense	c.2635G > A	p.E879K	VUS	8	rs774556391
8	Exon6	Frameshift deletion	c.596del	p.S199Wfs*122	LP	8	rs766103168
9	Exon21	Missense	c.2794G > A	p.D932N	VUS	6	rs147047438
10	Exon22	Missense	c.2921G > A	p.R974H	VUS	5	rs778216481
11	Exon32	Missense	c.4348T > C	p.Y1450H	LP	4	rs753591292
12	Exon12	Missense	c.1268C > T	p.T423I	VUS	3	rs201197899
13	Exon12	Missense	c.1310G > C	p.G437A	VUS	3	rs769796932
14	Exon14	Intronic	c.1693 + 10G > A	—	Benign	3	rs76411432
15	Exon15	Nonsense	c.1708C > T	p.Q570*	LP	3	rs1332668133
16	Exon20	Missense	c.2654G > A	p.R885Q	LP	3	rs181461079
17	Exon26	Nonframeshift deletion	c.3478_3480del	p.L1160del	LP	3	rs758318135
18	Exon5	Missense	c.364C > A	p.P122T	VUS	3	rs200265605
19	Exon10	Missense	c.1127G > T	p.R376L	LP	2	rs778729877
20	Exon11	Missense	c.1232G > A	p.R411K	VUS	2	rs764353021
21	Exon12	Missense	c.1294C > T	p.R432C	LP	2	rs978028019
22	Exon17	Nonframeshift deletion	c.2104_2106del	p.G702del	VUS	2	rs779340990
23	Exon4	Missense	c.244C > T	p.R82C	VUS	2	—
24	Exon26	Missense	c.3469C > T	p.L1157F	VUS	2	rs763092853
25	Exon28	Missense	c.3689C > T	p.A1230V	VUS	2	rs557220354
26	Exon29	Inframe insertion	c.3709_3711dup	p.S1237dup	VUS	2	rs766871326
27	Exon5	Missense	c.442G > C	p.V148L	VUS	2	rs764043878
28	Exon34	Missense	c.4567A > G	p.T1523A	VUS	2	rs149306918
29	Exon5	Missense	c.505C > T	p.R169W	VUS	2	rs201590426
30	Exon6	Missense	c.534G > C	p.W178C	LP	2	—
31	Exon9	Missense	c.959T > C	p.L320P	VUS	2	rs544236153
32	Exon6	Frameshift deletion	c.605_621del	p.Q202Rfs*93	LP	2	rs769318570
33	Exon10	Missense	c.1087A > G	p.S363G	VUS	1	—
34	Exon11	Missense	c.1172T > G	p.L391R	VUS	1	—
35	Exon3	Missense	c.127A > T	p.N43Y	VUS	1	rs747270555
36	Exon12	Nonsense	c.1300C > T	p.R434*	LP	1	rs119472026
37	Exon13	Missense	c.1462G > A	p.G488R	Pathogenic	1	rs191759494
38	Exon16	Nonsense	c.1921G > T	p.E641*	LP	1	—
39	Exon4	Missense	c.202G > T	p.G68C	VUS	1	rs886051198
40	Exon4	Missense	c.227C > T	p.P76L	LP	1	rs767705906
41	Exon18	Missense	c.2291G > A	p.R764Q	VUS	1	rs201884203
42	Exon18	Missense	c.2290C > T	p.R764W	VUS	1	rs141291775
43	Exon4	Missense	c.244C > A	p.R82S	LP	1	—
44	Exon19	Missense	c.2335G > A	p.V779M	VUS	1	rs145061993
45	Exon18	Missense	c.2275G > A	p.V759M	VUS	1	—
46	Exon18	Nonsense	c.2202G > A	p.W734*	LP	1	rs769789467
47	Exon21	Missense	c.2779A > G	p.M927V	VUS	1	rs755186335
48	Exon22	Missense	c.2894C > T	p.S965L	VUS	1	rs144153950
49	Exon25	Missense	c.3251G > A	p.R1084Q	VUS	1	rs558919433
50	Intron4	Intronic	c.326-20T > C	—	VUS	1	rs1209769448
51	Exon28	Missense	c.3632G > A	p.R1211H	VUS	1	rs141763307
52	Exon28	Missense	c.3667C > G	p.H1223D	VUS	1	rs374103492
53	Exon5	Missense	c.379G > A	p.E127K	VUS	1	rs760732287
54	Exon30	Missense	c.4015T > C	p.W1339R	VUS	1	—
55	Exon30	Missense	c.3902A > C	p.Q1301P	VUS	1	—
56	Exon30	Missense	c.4080G > T	p.K1360N	VUS	1	rs374891282
57	Exon33	Missense	c.4408C > T	p.R1470W	VUS	1	rs200785525
58	Exon5	Frameshift deletion	c.477del	p.E160Rfs*16	LP	1	rs1480917996
59	Exon6	Intronic splicing	c.514-1G > A	—	LP	1	—
60	Exon6	Intronic splicing	c.514-2A > G	—	LP	1	—
61	Exon5	Intronic	c.514-12C > A	—	VUS	1	rs754861497
62	Exon9	Missense	c.980A > C	p.E327A	VUS	1	—
63	Exon6	Frameshift insertion	c.602dup	p.Q202Tfs*99	LP	1	rs567500345

Variants were classified in accordance with the guidelines of the American College of Medical Genetics and Genomics (ACMG); —, absent in database; LP, likely pathogenic; VUS, variant of uncertain significance.

### Analysis of mutational outcomes and clinical phenotypes

**Figure 2:**
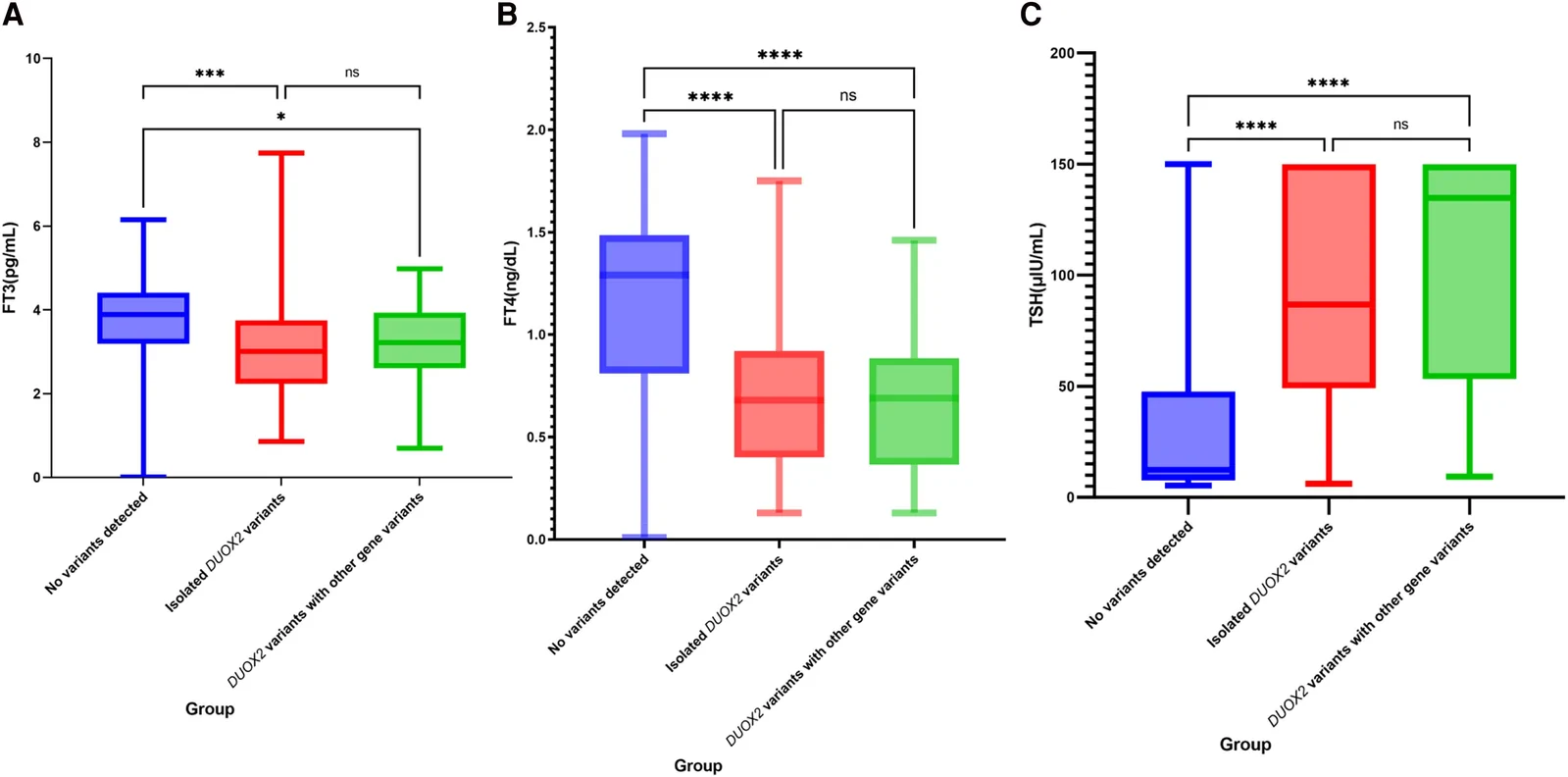
Comparison of thyroid function parameters at disease onset among 3 groups of subjects. (A) FT3 levels (pg/mL), (B) FT4 levels (ng/dL), (C) TSH levels (μIU/mL). Groups: No detected variants, isolated *DUOX2* variants, *DUOX2* variants combined with other gene variants. *P < .05; **P < .01; ***P < .001; ****P < .0001; ns, not significant.

Among 184 patients with CH, 34 cases with undetectable *DUOX2* mutations but carrying other types of genetic variants were excluded. The remaining patients were divided into 3 groups to investigate the correlation between *DUOX2* variants and clinical phenotypes. The 3 groups were as follows: the no detectable variants group, the isolated *DUOX2* variants group, and the *DUOX2* variants combined with other gene variants group (Table 2). There was no statistically significant difference in gender distribution among the 3 groups (*P* > .05). Thyroid ultrasound examinations in this study revealed that all children with thyroid hypoplasia exhibited isolated reduction in thyroid volume. And normal thyroid morphology was observed in the majority of children harboring *DUOX2* variants (Table 2). Notably, TSH levels were higher and FT4 and FT3 levels were lower in patients with the *DUOX2* variants compared with those without detectable variants (*P* < .05). However, no significant differences were observed in thyroid function indices between the isolated *DUOX2* variants group and the group with *DUOX2* variants combined with other variant types (Table 2, Fig. 2). These findings indicated that the hypothyroidism at onset was more severe in CH patients with *DUOX2* variants than in those without. Differences in treatment regimens and medication dosages between these groups will be further investigated in subsequent studies.

**Table 2:** Clinical, thyroid function test results and imaging features of CH patients positive or negative for *DUOX2* variants

	No variants detected (*n* = 70)	Isolated *DUOX2* variants (*n* = 43)	*DUOX2* variants with other gene variants (*n* = 37)	*P*-value
Sex				.246
Male	33 (47.14%)	26 (63.04%)	22 (62.22%)	
Female	37 (52.86%)	17 (36.96%)	15 (37.78%)	
Thyroid morphology at onset				.001
Gland in situ (normal)	47 (67.14%)*^ab^*	39 (90.70%)*^a^*	32 (86.49%)*^b^*	.016
Gland in situ (goitrous)	5 (7.14%)	1 (2.32%)	5 (13.51%)	.207
Thyroid dysgenesis (hypoplasia)	18 (25.72%)*^ab^*	3 (6.98%)*^a^*	0 (0%)*^b^*	<.001
Thyroid function at onset (median, IQR)				
FT3 (pg/mL)	3.85 (3.21-4.33)*^ab^*	3.68 (2.88-4.03)*^a^*	3.72 (3.41-4.03)*^b^*	<.0001
FT4 (ng/dL)	1.31 (1.13-1.49)*^ab^*	0.74 (0.39-1.41)*^a^*	0.75 (0.68-0.81)*^b^*	<.0001
TSH (μIU/mL)	8.99 (6.58-20.93)*^ab^*	52.45 (11.00-76.66)*^a^*	95.58 (90.43-100.73)*^b^*	<.0001

Abbreviation: IQR, interquartile range.

^*a*^Significant difference between patients with no variants detected group and isolated *DUOX2* variants group (*P* < .05).

^*b*^Significant difference between patients with no variants detected group and the *DUOX2* variants with other gene variants group (*P* < .05).

For the 80 children with *DUOX2* variants evaluated in this study, comprehensive clinical and genetic data are presented in Table S1 (27). Among these 80 children, 9 had abnormal results on their thyroid ultrasonography, which are detailed in Table 3. During the subsequent follow-up, 3 cases presented with growth and development retardation, and 1 case exhibited behavioral abnormalities (Table 4). Furthermore, no significant differences in clinical phenotypes were detected between children harboring only *DUOX2* variants and those with concomitant variants in other genes (*P* > .05).

**Table 3:** Clinical data and genetic variation spectrum analysis of patients with abnormal ultrasound findings related to *DUOX2* (*n* = 9)

Patient ID	Sex	Thyroid morphology	Thyroid function at onset			Mutation profile
			TSH (0.35-4.94)μIU/mL	FT4 (0.89-1.76)ng/dL	FT3 (2.30-4.20)pg/mL	
FJCHT0094	Female	Goiter	>150.00	0.35	1.62	DUOX2(c.3693 + 1G > T;c.3478_3480del/p.L1160del); GBP1(c.560T > C/p.L187P)
FJCHT0240	Male	Goiter	143.26	0.49	2.55	DUOX2(c.1588A > T/p.K530*;c.1300C > T > p.R434*); SLC26A7(c.1576A > G/p.M526V)
FJCHT0256	Female	Goiter	16.64	0.77	4.98	DUOX2(c.2048G > T/p.R683L;c.2794G > A/p.D932N;c.4027C > T/p.L1343F); DUOXA1(c.937A > G/p.S313G); SHH(c.869G > A/p.G290D); SLC26A4(c.757A > G/p.I253V)
FJCHT0258	Female	Goiter	57.42	0.85	4.48	DUOX2(c.3693 + 1G > T;c.1232G > A/p.R411K);GLIS3(c.2723C > T/p.A908V);TSHR(c.733G > A/p.G245S)
FJCHT0259	Male	Goiter	>150.00	0.13	1.24	DUOX2(c.1268C > T/p.T423I;c.3329G > A/p.R1110Q);DUOXA1(c.638C > T/p.T213M);TG(c.3908G > A/p.G1303D)
FJCHT0002	Male	Goiter	147.00	0.24	1.88	DUOX2(c.3693 + 1G > T;c.364C > A/p.P122T)
FJCHT0325	Female	Hypoplasia	>150.00	0.61	3.14	DUOX2(c.1310G > C/p.G437A;c.1127G > T/p.R376L)
FJCHT0329	Male	Hypoplasia	86.79	0.80	3.55	DUOX2(c.1588A > T/p.K530*)
FJCHT0378	Male	Hypoplasia	6.13	1.75	3.72	DUOX2(c.3478_3480del/p.L1160del;c.1588A > T/p.K530*)

**Table 4:** Clinical data and genetic variant profiles in patients with abnormal growth and development (*n* = 4)

Patient ID	Sex	Thyroid morphology	Thyroid function at onset			Growth and Development Status	Intellectual Condition	Mutation profile
			TSH (0.35-4.94)μIU/mL	FT4 (0.89-1.76)ng/dL	FT3 (2.30-4.20)pg/mL			
FJCHT0002	Male	Goiter	147.00	0.24	1.88	Slow growth	Generally normal	DUOX2(c.3693 + 1G > T;c.364C > A/p.P122T)
FJCHT0017	Female	Gland in situ (Normal)	9.18	1.31	4.00	Slow growth	Generally normal	DUOX2(c.3693 + 1G > T;c.2654G > A/p.R885Q)
FJCHT0322	Female	gland in situ (normal)	66.73	0.23	1.04	Slow growth	Generally normal	DUOX2(c.1588A > T/p.K530*)
FJCHT0411	Male	Gland in situ (normal)	8.37	1.30	5.03	Abnormal behavior	Generally normal	DUOX2(c.4027C > T/p.L1343F;c.2048G > T > T/p.R683L;c.1693 + 10G > A)

### In vitro functional analysis of the enzymatic activity of DUOX2 variants

**Figure 3:**
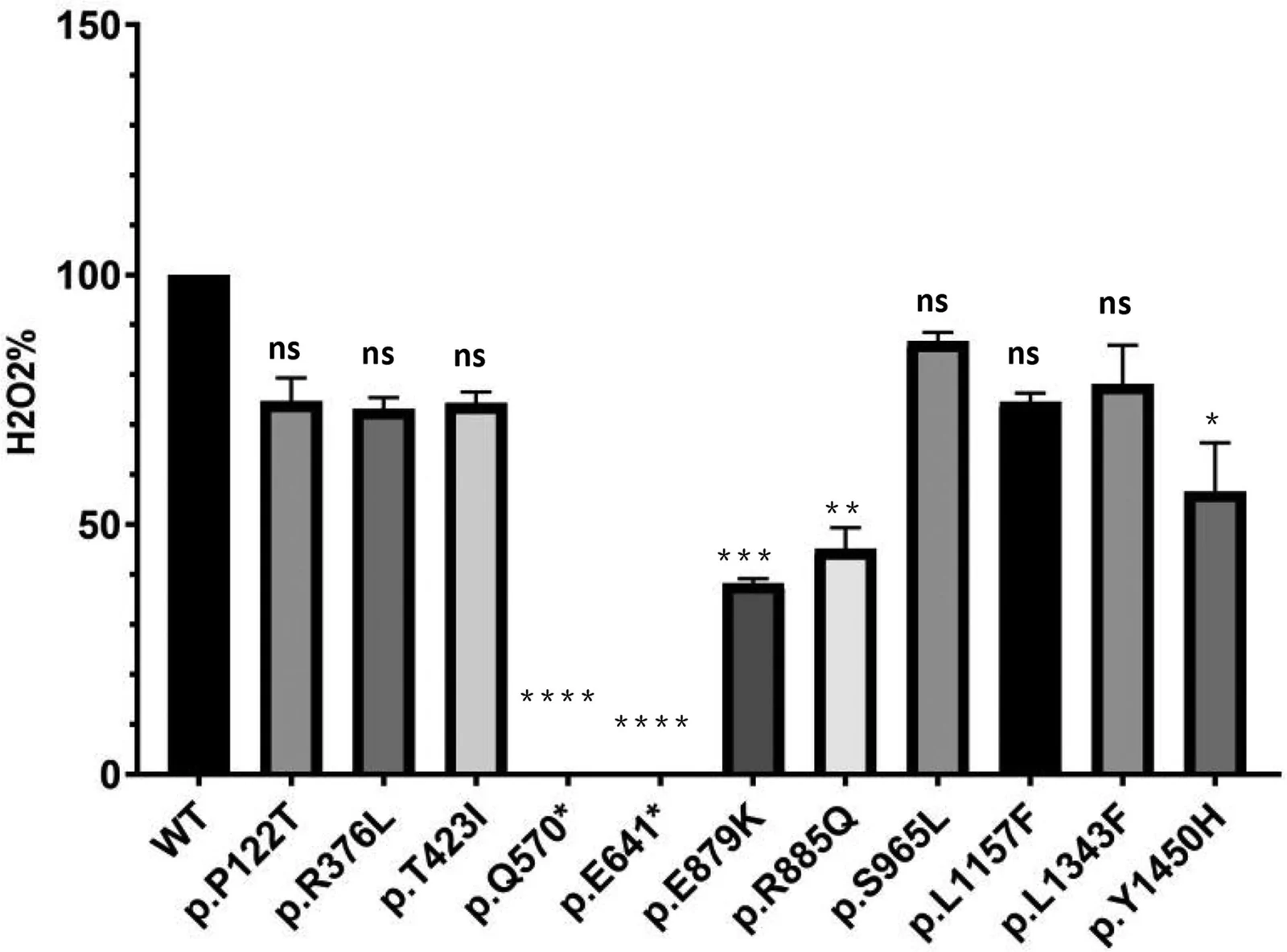
Enzymatic activity analysis of 11 *DUOX2* variants by H_2_O_2_ production assay. The capacity of DUOX2 protein to generate H_2_O_2_ was measured using Amplex Red reagent. the enzymatic activity of each *DUOX2* variant was normalized to that of WT (set as 100%), with mock transfection as the negative control (set as 0%). All experiments were performed in triplicate, and the results are presented as mean ± SD. Pathogenicity classification of each mutation type is as follows: p.P122T (VUS), p.R376L (LP), p.T423I (VUS), p.Q570* (LP), p.E641* (LP), p.E879 K (VUS), p.R885Q (LP), p.S965L (VUS), p.L1157F (VUS), p.L1343F (LP), p.Y1450H (LP). *P < .05; **P < .01; ***P < .001; ****P < .0001; ns, not significant.

The 11 functional variants selected in this study were prioritized through comprehensive evaluation of the study cohort, encompassing high-frequency recurrent variants, nonsense truncating variants, and low-frequency variants with clinically representative characteristics. Through in vitro enzymatic activity assessment, we explored how these domain-specific variants affect enzyme function, with the aim of further confirming their pathogenicity (Fig. 1). Compared with the WT control, 5 variants (p.K570*, p.E641*, p.E879K(VUS), p.R885Q, and p.Y1450H) exhibited statistically significant reductions in enzymatic activity (*P* < .05) (Fig. 3). All experiments were performed in triplicate, and the results are presented as mean ± SD. Notably, not all observed alterations in enzymatic function were confined to the dual peroxidase-like domain as initially anticipated; variants located in other domains and nonstructural regions also significantly affected enzyme function. For example, the p.E641* variant, located in a nonstructural region, showed a significant reduction in enzyme activity to approximately 14% of the WT level (*P* < .0001). In contrast, p.T423I(VUS) variant, which resides within the peroxidase functional domain, retained approximately 74.35% of its normal enzymatic activity (*P* > .05). Although the p.L1343F variant is widely recognized as a pathogenic variant (9), its residual enzymatic activity remained as high as 78.23% of the WT level (*P* > .05). This observation may be attributed to the functional threshold of the gene product, the genetic pattern of the variant (eg, homozygous vs compound heterozygous), and the coexistence of other synergistic variants (28). The 3 novel variants reported herein have not been previously described in the literature. Owing to the lack of familial segregation data and definitive clinical correlation, these variants were excluded from the functional experimental cohort in the present study.

## Discussion

In the present study, we performed exome sequencing to examine the mutation spectrum of CH-associated genes in 184 children with CH from Fujian Province, China. *DUOX2* variants were the most commonly detected, accounting for 43.48% (80/184) of patients. Our results confirm that *DUOX2* is the major pathogenic gene for CH in the Chinese population, consistent with previous reports from East Asian cohorts (7-10). The high prevalence of *DUOX2* variants in our study underscores its important role in the pathogenesis of CH in Fujian, and provides useful epidemiological data for the regional prevention and clinical management of CH. We identified 63 different *DUOX2* variants, including 3 novel variants: c.980A > C (p.E327A), c.4015T > C (p.W1339R), and c.1172T > G (p.L391R). These new findings extend the currently known genetic spectrum of CH. The most common *DUOX2* variants in our cohort, c.1588A > T (p.K530*), c.3329G > A (p.R1110Q), and c.4027C > T (p.L1343F), have also been reported in earlier Chinese studies (9, 10), suggesting that these variants may represent founder mutations in the Han Chinese population. *Dual oxidase 2* variants were distributed across multiple functional domains, including the dual peroxidase-like domain, EF-hand domain, and ferric oxidoreductase domain. This broad distribution implies that structural or functional damage in any of these regions may compromise DUOX2 activity, which further supports the essential function of *DUOX2* in thyroid hormone biosynthesis (12, 17).

We systematically analyzed the clinical phenotypes of patients harboring *DUOX2* variants. The analysis covered thyroid function indices, thyroid ultrasonography characteristics, physical growth, and neurodevelopmental outcomes. Compared with CH patients without identifiable variants, those harboring *DUOX2* variants exhibited significantly elevated TSH levels, along with markedly reduced FT4 and FT3 levels. Interestingly, no significant differences in clinical phenotypes were observed between children with isolated *DUOX2* variants and those with concomitant variants in additional genes. These findings indicate that *DUOX2* variants are strongly associated with impaired thyroid hormone synthesis and correlate closely with the initial clinical presentation at diagnosis. Thyroid hormone levels correlate closely with treatment outcomes in CH patients. Variations in thyroid hormone levels during therapy may reflect the functional characteristics of *DUOX2* variants, suggesting their potential as biomarkers for monitoring treatment response. CH exhibits substantial phenotypic heterogeneity influenced by genetic penetrance, concurrent gene mutations, and environmental factors. The mechanistic link between *DUOX2* variants and clinical response therefore remains poorly defined. Future large-cohort long-term follow-up will integrate longitudinal thyroid function indices and clinical symptom changes to clarify this association and validate the clinical utility of *DUOX2* variants as biomarkers. Furthermore, the study demonstrated that patients harboring *DUOX2* variants presented with more severe hypothyroidism at initial diagnosis, whereas the majority exhibited a morphologically normal thyroid gland on ultrasonography. However, a previous study demonstrated that some patients with biallelic *DUOX2* variants presented with transient hypothyroidism and could discontinue levothyroxine treatment after 3 years of age while preserving normal thyroid function (14). This observation appears to be inconsistent with the current findings and suggests that compensatory mechanisms may exist to promote thyroid follicular cell proliferation and goiter over time. Future investigations are warranted to explore whether thyroid cell-related stimulatory antibodies or other proliferative factors are present and contribute to thyroid tissue growth and functional recovery. Elucidating such mechanisms may provide a potential strategy to improve the rate of successful treatment withdrawal in selected patients.

Our in vitro findings demonstrated that not all genetic variants within functional domains significantly impact enzymatic activity. The minimal effects of p.P122T, p.R376L, p.T423I, p.L1157F, and p.L1343F variants suggest that the development of CH may not solely depend on reduced enzymatic activity. Alternative pathogenic mechanisms may include the impaired trafficking of the DUOX2 protein to the cell membrane which directly disrupts thyroid hormone synthesis (29) or decreased protein stability due to shortened half-life and accelerated degradation (16). Notably, this study only evaluated enzymatic activity for the functional characterization of *DUOX2* variants, without systematically assessing other functional attributes (eg, protein expression levels, and stability). Future studies should refine experimental designs to comprehensively evaluate the pathogenic potential of *DUOX2* variants. The variants located in unstructured domain (eg, p.E641*) significantly reduced enzymatic activity. We hypothesize that variants in unstructured regions may interfere with the proper folding of DUOX2 protein. Correct folding is a critical step for forming a stable complex with its maturation factor, DUOXA2, at the cell surface, which is essential for H_2_O_2_ production. While this hypothesis requires further experimental validation. A recent study on a novel *DUOXA2* missense mutation demonstrated that *DUOXA2* variants can impair NADPH oxidase activity without altering protein expression, further confirming the functional interdependence of the DUOX2 and DUOXA2 protein in thyroid hormone synthesis (30). Previous studies have reported a significant association between residual DUOX2 enzymatic activity and clinical phenotype at diagnosis (7). However, this study only analyzed the enzymatic activity of 11 *DUOX2* variant types, no such correlation was observed. This discrepancy is likely due to the limited number of variants analyzed. Further systematic research is therefore required, including comprehensive assessment of enzymatic activity across expanded variant loci and larger patient cohorts. However, functional experiments still lack unified standardized protocols—especially for variants of VUS—which is essential to improve the accuracy of pathogenicity interpretation for these variants.

This study makes several incremental contributions to the research field. By focusing on Han Chinese children with CH, it supplements population-specific genetic data regarding *DUOX2* variants and enriches the variant spectrum across different ethnic groups. Three previously undescribed variants were uncovered, expanding the current repertoire of the *DUOX2*. Functional enzymatic activity profiling of 11 key variants delineated their molecular characteristics, furnishing direct experimental evidence for genotype–phenotype correlation and endowing the present work with greater clinical translational value relative to investigations confined solely to variant screening. Combined clinical data further enabled a preliminary elucidation of the relationship between *DUOX2* alterations and phenotypic manifestations, providing valuable insights to inform precision diagnosis and individualized clinical intervention for affected patients.

Our study has several limitations that should be acknowledged. First, the limited number of *DUOX2* variants evaluated for enzymatic activity may have hindered our ability to detect a statistically significant correlation between residual activity and clinical phenotype. Second, we did not assess other functional properties of *DUOX2* variants (eg, protein trafficking and stability), which may contribute to their pathogenicity. Finally, the follow-up duration was not standardized, precluding a comprehensive analysis of long-term clinical outcomes including treatment response.

## Conclusion

In conclusion, *DUOX2* variants represent the most common genetic cause of CH in children from Fujian Province. Our study further revealed that variants in other genes can coexist with *DUOX2* variants, contributing to the complex genetic etiology of CH. Compared with patients without identifiable pathogenic variants, those carrying *DUOX2* variants exhibited significantly elevated TSH levels, and markedly reduced FT4 and FT3 levels. However, no clear correlation was observed between clinical phenotypes and specific mutation types. Furthermore, no significant phenotypic differences were found between patients with isolated *DUOX2* variants and those with additional concomitant variants. This may be attributed to the high sensitivity of TSH screening, which enables the early identification and timely treatment of affected children before more severe phenotypes develop. In vitro functional analysis shows that *DUOX2* variants impair H_2_O_2_ generation, with variants in both structured and unstructured domains exerting pathogenic effects. Due to the limited number of variants analyzed in this study, a robust correlation between residual enzyme activity and clinical severity could not be established. Future studies should expand the functional validation cohorts and comprehensive assessments, such as protein half-life and stability, to more accurately determine the pathogenicity of *DUOX2* variants. Such integrative functional approaches are essential for strengthening the scientific validity and clinical utility of genetic counseling.

## Data Availability

Some or all datasets generated during and/or analyzed during the current study are not publicly available but are available from the corresponding author on reasonable request. The supplementary tables that support the findings of this study are available in the Figshare repository at http://doi.org/10.6084/m9.figshare.32258151.

## Clinical trial information

ChiCTR ChiCTR26000129068; https://www.chictr.org.cn.

## References

[1] Mengreli C, Kanaka-Gantenbein C, Girginoudis P, et al Screening for congenital hypothyroidism: the significance of threshold limit in false-negative results. J Clin Endocrinol Metab. 2010;95(9):4283‐4290. 20591982 10.1210/jc.2010-0057

[2] van Trotsenburg P, Stoupa A, Léger J, et al Congenital hypothyroidism: a 2020-2021 Consensus Guidelines Update-An ENDO-European Reference Network Initiative Endorsed by the European Society for Pediatric Endocrinology and the European Society for Endocrinology. Thyroid. 2021;31(3):387‐419. 33272083 10.1089/thy.2020.0333 PMC8001676

[3] Han B, Sun F, He Y, et al Interpretation of the 2025 “Guidelines for the diagnosis and treatment of congenital hypothyroidism”. Zhonghua Yi Xue Za Zhi. 2025;105(47):4345‐4350. Chinese. 41429496 10.3760/cma.j.cn112137-20250811-02036

[4] Löf C, Patyra K, Kuulasmaa T, et al Detection of novel gene variants associated with congenital hypothyroidism in a Finnish patient cohort. Thyroid. 2016;26(9):1215‐1224. 27373559 10.1089/thy.2016.0016 PMC5036323

[5] Nicholas AK, Serra EG, Cangul H, et al Comprehensive screening of eight known causative genes in congenital hypothyroidism with gland-in-situ. J Clin Endocrinol Metab. 2016;101(12):4521‐4531. 27525530 10.1210/jc.2016-1879 PMC5155683

[6] Kurnaz E, Türkyılmaz A, Yaralı O, Dönmez AS, Çayır A. Genetic analyses in a Cohort of pediatric patients with congenital hypothyroidism based on congenital hypothyroidism consensus guideline. Horm Res Paediatr. 2026;99(1):23‐37. 39378853 10.1159/000541898 PMC12795535

[7] Sorapipatcharoen K, Tim-Aroon T, Mahachoklertwattana P, et al DUOX2 variants are a frequent cause of congenital primary hypothyroidism in Thai patients. Endocr Connect. 2020;9(11):1121‐1134. 33310921 10.1530/EC-20-0411 PMC7774760

[8] Tanaka T, Aoyama K, Suzuki A, Saitoh S, Mizuno H. Clinical and genetic investigation of 136 Japanese patients with congenital hypothyroidism. J Pediatr Endocrinol Metab. 2020;33(6):691‐701. 32469330 10.1515/jpem-2019-0433

[9] Sun F, Zhang RJ, Cheng F, et al Correlation of DUOX2 residual enzymatic activity with phenotype in congenital hypothyroidism caused by biallelic DUOX2 defects. Clin Genet. 2021;100(6):713‐721. 34564849 10.1111/cge.14065

[10] Ahn J, Jeong H. Genetic Etiology of permanent congenital hypothyroidism in Korean patients: a whole-exome sequencing study. Int J Mol Sci. 2025;26(9):4465. 40362701 10.3390/ijms26094465 PMC12072708

[11] Wang F, Zang Y, Li M, et al *DUOX2* and *DUOXA2* variants confer susceptibility to thyroid dysgenesis and gland-*in-situ* with congenital hypothyroidism. Front Endocrinol (Lausanne). 2020;11:237. 32425884 10.3389/fendo.2020.00237 PMC7212429

[12] Molina MF, Papendieck P, Sobrero G, et al Mutational screening of the TPO and DUOX2 genes in Argentinian children with congenital hypothyroidism due to thyroid dyshormonogenesis. Endocrine. 2022;77(1):86‐101. 35507000 10.1007/s12020-022-03054-3

[13] Vincenzi G, Petralia IT, Abbate M, Vigone MC. 50 YEARS OF NEWBORN SCREENING FOR CONGENITAL HYPOTHYROIDISM: EVOLUTION OF INSIGHTS IN ETIOLOGY, DIAGNOSIS AND MANAGEMENT: transient or permanent congenital hypothyroidism: from milestones to current and future perspectives. Eur Thyroid J. 2025;14(4):e250019. 40686339 10.1530/ETJ-25-0019 PMC12330207

[14] Sun F, Wan JP, Zhang NN, et al Clinical outcomes of congenital hypothyroidism due to *DUOX2* biallelic mutations after levothyroxine withdrawal. Thyroid. 2025;35(10):1120‐1128. 40916794 10.1177/10507256251372195

[15] Dermitzaki N, Serbis A, Baltogianni M, Balomenou F, Giapros V. Permanent or transient congenital hypothyroidism: a diagnostic dilemma. Acta Paediatr. 2026;115(1):43‐54. 40976938 10.1111/apa.70312 PMC12687691

[16] Liu S, Zhang W, Zhang L, et al Genetic and functional analysis of two missense *DUOX2* mutations in congenital hypothyroidism and goiter. Oncotarget. 2018;9(4):4366‐4374. 29435108 10.18632/oncotarget.10525 PMC5796979

[17] De Deken X, Miot F. DUOX defects and their roles in congenital hypothyroidism. Methods Mol Biol. 2019;1982:667‐693. 31172499 10.1007/978-1-4939-9424-3_37

[18] Fu C, Luo S, Zhang S, et al Next-generation sequencing analysis of DUOX2 in 192 Chinese subclinical congenital hypothyroidism (SCH) and CH patients. Clin Chim Acta. 2016;458:30‐34. 27108200 10.1016/j.cca.2016.04.019

[19] Zheng Z, Yang L, Sun C, et al Genotype and phenotype correlation in a cohort of Chinese congenital hypothyroidism patients with DUOX2 mutations. Ann Transl Med. 2020;8(24):1649. 33490161 10.21037/atm-20-7165 PMC7812163

[20] Su Y, Lei X, Muhetaer A, He J, Li L. Correlation of genotype-phenotype of congenital hypothyroidism cohort diagnosed by newborn screening: a long-term observational study. Int J Neonatal Screen. 2025;11(4):98. 41133710 10.3390/ijns11040098 PMC12550986

[21] Yang RM, Zhan M, Zhou QY, et al Upregulation of GBP1 in thyroid primordium is required for developmental thyroid morphogenesis. Genet Med. 2021;23(10):1944‐1951. 34194003 10.1038/s41436-021-01237-3 PMC8486662

[22] Richards S, Aziz N, Bale S, et al Standards and guidelines for the interpretation of sequence variants: a joint consensus recommendation of the American College of Medical Genetics and Genomics and the Association for Molecular Pathology. Genet Med. 2015;17(5):405‐424. 25741868 10.1038/gim.2015.30 PMC4544753

[23] Rurale G, Marelli F, Duminuco P, Persani L. *Glis3* as a critical regulator of thyroid primordium specification. Thyroid. 2020;30(2):277‐289. 31797737 10.1089/thy.2019.0196

[24] Yamaguchi T, Nakamura A, Nakayama K, et al Targeted next-generation sequencing for congenital hypothyroidism with positive neonatal TSH screening. J Clin Endocrinol Metab. 2020;105(8):dgaa308. 32459320 10.1210/clinem/dgaa308

[25] Kang HS, Grimm SA, Jothi R, Santisteban P, Jetten AM. GLIS3 regulates transcription of thyroid hormone biosynthetic genes in coordination with other thyroid transcription factors. Cell Biosci. 2023;13(1):32. 36793061 10.1186/s13578-023-00979-8 PMC9930322

[26] Zhang HY, Zhang CX, Wu FY, et al Large-scale screening and functional study of DUOXA2 variant in 599 Chinese patients with congenital hypothyroidism. Eur J Endocrinol. 2025;193(1):21‐30. 40510014 10.1093/ejendo/lvaf123

[27] Huang S-H, Sun F, Luo F, et al Supplementary data for Genetic Screening and Functional Characterization of DUOX2 Mutations in Patients with Congenital Hypothyroidism. Figshare; 2026. 10.6084/m9.figshare.32258151 PMC13519044 42662067

[28] Fu C, Xie B, Zhang S, et al Mutation screening of the TPO gene in a cohort of 192 Chinese patients with congenital hypothyroidism. BMJ Open. 2016;6(5):e010719. 10.1136/bmjopen-2015-010719 PMC4874165 27173810

[29] Grasberger H, De Deken X, Miot F, Pohlenz J, Refetoff S. Missense mutations of dual oxidase 2 (DUOX2) implicated in congenital hypothyroidism have impaired trafficking in cells reconstituted with DUOX2 maturation factor. Mol Endocrinol. 2007;21(6):1408‐1421. 17374849 10.1210/me.2007-0018

[30] Liu S, Liu L, Niu X, Lu D, Xia H, Yan S. A novel missense mutation (I26M) in DUOXA2 causing congenital goiter hypothyroidism impairs NADPH oxidase activity but not protein expression. J Clin Endocrinol Metab. 2015;100(4):1225‐1229. 25675383 10.1210/jc.2014-3964

